# Modeling Impacts of Urbanization on Winter Boundary Layer Meteorology and Aerosol Pollution in the Central Liaoning City Cluster, China

**DOI:** 10.3390/toxics11080683

**Published:** 2023-08-09

**Authors:** Dongdong Wang, Yangfeng Wang, Xiaolan Li, Lidu Shen, Chenhe Zhang, Yanjun Ma, Ziqi Zhao

**Affiliations:** 1 Institute of Atmospheric Environment, China Meteorological Administration, Shenyang 110166, China; wangdongdong@iaesy.cn (D.W.);; 2 Key Opening Laboratory for Northeast China Cold Vortex Research, Shenyang 110166, China; 3 Collaborative Innovation Center of Atmospheric Environment and Equipment Technology, Jiangsu Key Laboratory of Atmospheric Environment Monitoring and Pollution Control (AEMPC), Nanjing University of Information Science & Technology, Nanjing 210044, China; 4 Institute of Applied Ecology, Chinese Academy of Sciences, Shenyang 110016, China; 5 Liaoning Meteorological Observatory, Shenyang 110166, China

**Keywords:** urbanization, land-use change (LU), urban canopy (UC), WRF–Chem, boundary layer meteorology, PM_2.5_, Central Liaoning city cluster (CLCC), atmospheric diffusion capacity (ADC)

## Abstract

The influence of urbanization on the frequent winter aerosol pollution events in Northeast China is not fully understood. The Weather Research and Forecasting Model with Chemistry (WRF–Chem) coupled with urban canopy (UC) models was used to simulate the impact of urbanization on an aerosol pollution process in the Central Liaoning city cluster (CLCC), China. To investigate the main mechanisms of urban expansion and UC on the winter atmospheric environment and the atmospheric diffusion capacity (ADC) in the CLCC, three simulation cases were designed using land-use datasets from different periods and different UC schemes. A comparative analysis of the simulation results showed that the land-use change (LU) and both LU and UC (LUUC) effects lead to higher surface temperature and lower relative humidity and wind speed in the CLCC by decreasing surface albedo, increasing sensible heat flux, and increasing surface roughness, with a spatial distribution similar to the distribution of LU. The thermal effect leads to an increase in atmospheric instability, an increase in boundary layer height and diffusion coefficient, and an increase in the ADC. The LU and LUUC effects lead to a significant decrease in near-surface PM_2.5_ concentrations in the CLCC due to changes in meteorological conditions and ADC within the boundary layer. The reduction in surface PM_2.5_ concentrations due to the LU effect is stronger at night than during daytime, while the LUUC effect leads to a greater reduction in surface PM_2.5_ concentrations during the day, mainly due to stronger diffusion and dilution caused by the effect of urban turbulence within different levels caused by the more complex UC scheme. In this study, the LU and LUUC effects result in greater thermal than dynamic effects, and both have a negative impact on surface PM_2.5_ concentrations, but redistribute pollutants from the lower urban troposphere to higher altitudes.

## 1. Introduction

China has experienced very rapid development and urbanization in recent decades [1]. The urbanization process is considered an extreme example of land-use change (LU) caused by human activities [1,2,3]. Urban expansion through the transformation of natural vegetation into artificial constructions and the formation of urban canopies by high-rise complexes are key features of the urbanization process, which affects the dynamic and thermal properties of the underlying surface, such as the impervious surface, roughness, albedo, and heat flux [4,5]. Changes in these characteristics directly lead to changes in the urban canopy (UC) structure, which affects the surface energy balance [6,7] and local atmospheric circulation [8], further affecting air quality in the urban boundary layer [9,10,11,12,13,14] and generating catastrophic meteorological events, such as hot summer heat waves [1]. These can have adverse effects on human and ecological health [15,16,17,18]. Therefore, the external forcing on the atmospheric environment due to urbanization development, especially the change of underground characteristics, is a hot topic of current research in the field of the atmospheric environment [12].

The research results indicate that changes in pollutant distribution are sensitive to urban expansion [1,8,11,12]. Numerical simulation using advanced chemical climate models combined with urban land modules has become an applicable method to estimate the impact of urbanization [1,19,20]. Previous numerical studies have focused on the effects of urbanization on urban meteorological fields [7,21,22,23], while others have focused on the impact of urbanization-induced meteorological changes on air quality [1,5,9,13,24,25]. The impact of urbanization on PM_2.5_ is very complicated [14]. Liu et al. [26] suggested that the warming and high surface roughness in urban areas reduce the horizontal wind speed at ground level, which inhibits the outflow of air pollutants and leads to an increase in PM_2.5_. In contrast, other studies have shown that urbanization-induced warming reduces surface aerosol concentration by enhancing turbulent mixing in the deeper urban boundary layer [1,10,12,27,28]. From the perspective of urbanization-induced meteorological effects on pollution diffusion, it is mainly divided into thermal effect and dynamic effect. The thermal effect refers to the urban heat island effect on pollution diffusion caused by the urban LU and anthropogenic heat release, and the dynamic effect is the dragging effect of urban buildings on airflow, which causes the urban wind speed and the ADC to decrease. In addition, the UC schemes have a significant impact on the simulation results of urban meteorological conditions and atmospheric environments, and different UC schemes are crucial for the simulation of boundary layers and near-surface meteorological fields. Several studies have shown that the simulated results of the multilayer UC schemes are more consistent with actual observation [29,30,31,32].

Previous numerical modeling of the impact of urbanization in China has focused on developed regions, such as the Yangtze River Delta [10,11,14,33,34], Pearl River Delta [8,12,13,19,25], and Beijing–Tianjin–Hebei [1], with little analysis of the impact on cities in Northeast China, particularly in the Central Liaoning city cluster (CLCC) [35]. The CLCC comprises five cities: Shenyang (SY), Anshan (AS), Fushun (FS), Benxi (BX), and Liaoyang (LY), which are the fastest urbanizing areas in Liaoning Province in recent years, are densely populated, and play a very important role in the development of Liaoning and even Northeast China. Studies have shown the frequent occurrence of severe aerosol pollution in the CLCC, which is closely related to high anthropogenic emissions and unfavorable diffusion conditions due to strong stability [36,37,38,39,40]. However, it is not yet clear how the urbanization process affects winter aerosol pollution in the CLCC. Therefore, it is necessary to investigate the effects and mechanisms of urbanization in the CLCC.

In this study, we focused on a winter PM_2.5_ pollution event in the CLCC and investigated the impact of urbanization-induced LU and both LU and UC (LUUC) effects on the changes of ADC and PM_2.5,_ as well as the mechanisms involved. The results are expected to provide a reference for an in-depth study of the effects of rapid urbanization on weather and air quality, the mechanisms of which could provide an important theoretical basis for rational planning of urban development, and economic and environmental decisions. The remainder of this paper is organized as follows: Section 2 introduces the configurations of the air quality model, and observational data are described. Section 3 presents the main results, and the main findings are summarized in Section 4.

## 2. Materials and Methods

### 2.1. Materials

**Figure 1:**
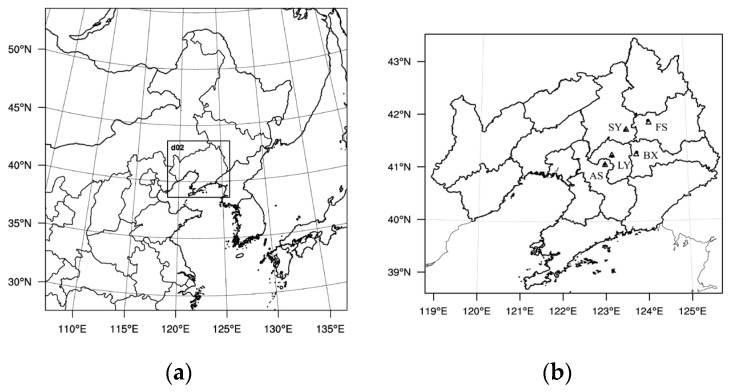
(**a**) Double-nested modeling domains and (**b**) the locations of five urban meteorological stations (black dots) and environmental monitoring stations (triangles) in the CLCC.

The Weather Research and Forecasting Model (WRF) with Chemistry (WRF–Chem) version 3.9.1 was used to better understand the effects of urban expansion and UC on the regional atmospheric environment due to urbanization in the CLCC [41]. The simulation adopts two layers of nesting, with the innermost simulated area being the Liaoning Province area (Figure 1). The number of nested grids in the two layers is 322 × 322 and 199 × 181, respectively, and the horizontal resolution is 9 km and 3 km, respectively. The model outputs results once every 1 h. The top of the model is set at 50 hPa and is divided vertically into 49 layers, 10 of which are within 1.5 km near the surface and 3 of which are within 100 m near the surface. Model physical process schemes include the Lin microphysics [42,43], the RRTMG long- and shortwave radiation scheme [44], the Noah land surface parameterization scheme [45], the MYJ boundary layer scheme [46,47], and the Monin Obukhov (Janjić Eta) near-stratigraphic scheme [48]. The chemical process uses the CBMZ meteorological chemistry mechanism [49] and the MOSAIC aerosol chemistry scheme [50,51]. The photolysis mechanism is calculated online using the Fast-J method [52]. The fifth-generation European Centre for Medium-Range Weather Forecasts reanalysis (ERA5) dataset with a spatial resolution of 0.25° × 0.25° and a temporal resolution of 6 h (https://cds.climate.copernicus.eu/cdsapp#!/dataset/reanalysis-era5-pressure-levels?tab=overview, and https://cds.climate.copernicus.eu/cdsapp#!/dataset/reanalysis-era5-single-levels?tab=overview, accessed on 1 August 2023) was used to adopt the initial meteorological field and boundary conditions for WRF–Chem model simulations. The output data from the CAM–Chem global model was used as the initial and boundary condition for the chemical fields (https://www.acom.ucar.edu/cam-chem/cam-chem.shtml, accessed on 1 August 2023). The gridded dataset of emission sources uses the 2016 Multi-resolution Emission Inventory of China (MEIC; http://meicmodel.org.cn/, accessed on 1 August 2023) with a spatial resolution of 0.25° × 0.25° [53,54]. Emissions from all species include four sectors (electricity, industry, residential, and transport) [1,13]. The emission sources were spatially assigned to grid cells with horizontal resolutions of 9 km and 3 km. Although the coarse resolution of the emission inventories affects the simulation results to some extent, this effect is not significant for this study. The simulation period was selected from 30 December 2019 to 7 January 2020, and the main study period for the pollution process was 1–5 January 2020, considering the model spin-up time (16 h). Each experiment was analyzed using the second level of nested results (3 km). The software used to draw the map in this paper was NCL Version 6.2.1 (National Center for Atmospheric Research NCAR, Boulder, CO, USA) [55].

The WRF–Chem model includes both United States Geological Survey (USGS) and Moderate Resolution Imaging Spectroradiometer (MODIS) land-use data set, which represent 1992/1993 and 2004 land-use conditions, respectively [56]. However, both land-use data have lagged behind the increasing expansion of urban areas. Therefore, the MODIS 2018 land-use data was introduced in this study, which can clearly reflect the current urbanization process in the Liaoning Province. The models used a single-layer UCM [57,58] and a multilayer building energy parameterization (BEP) UCM [32,59] in separate comparative experiments to examine the effects of UC. In order to investigate the role of urban expansion and UC on the regional atmospheric environment, three simulations were set up in this study, using the same physical and chemical schemes, initial and boundary conditions, and chemical emission source inventory, differing only in the information on LU data set and UC scheme, as detailed in Table 1. 

**Figure 2:**
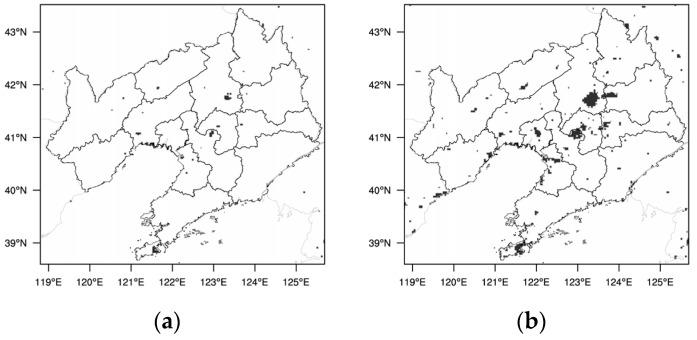
The distribution of the Liaoning urban area from different land-use data sets, (**a**) USGS, (**b**) MODIS 2018.

The USGS data can represent the situation without large-scale urbanization, with small urban areas and fragmented distribution (Figure 2a). The MODIS 2018 data can represent the LU conditions after large-scale urbanization (Figure 2b). By comparison, it can be seen that the Liaoning Province experienced rapid urban expansion from the 1990s to the 2010s, with a significant increase in urban land area, especially in the CLCC. The transformation of LU types is mainly from forest, farmland, etc., to urban land, similar to the situation in some other provines and cities [1,8,12,19,56]. In the analysis, the effects of LU and LUUC can be derived from the difference between the M-B and U-B simulations and the difference between the M-B and U-U simulations, respectively.

### 2.2. Observation Data

The hourly surface meteorological observation data were obtained from the National Meteorological Center (http://data.cma.cn/, accessed on 1 August 2023), including temperature, humidity, and wind speed. The hourly dataset of PM_2.5_ mass concentrations and daily AQI were obtained from the China Air Quality Online Monitoring and Analysis Platform (https://www.aqistudy.cn/, accessed on 1 August 2023), and the environmental monitoring stations were selected to be closest to the meteorological stations. The locations of meteorological observation and environmental monitoring stations are shown in Figure 1b. 

### 2.3. Model Validation

To test the simulation capability and reliability of the WRF–Chem model, the model results were verified with ground-based meteorological observations and PM_2.5_ concentration monitoring data from Shenyang (SY) and the CLCC. The model results are hourly output values interpolated to the station locations of five cities (Figure 1b). The following statistical indicators were used to evaluate the consistency and accuracy of the simulation results with the observed data: mean bias (MB), general error (GE), root mean square error (RMSE), correlation coefficient (R), and index of agreement (IOA), respectively. MB and GE reflect the degree of deviation between simulated and observed values, and RMSE evaluates the difference between simulated and observed data, which can reflect the simulation effect on extreme conditions. The closer the value of each parameter is to 0, the better the simulation effect. R and IOA are chosen to characterize the degree of agreement of the trend between the simulated and observed values, with values closer to 1 indicating better simulation. The calculation formulas for each indicator refer to references [14,60].

Observations and simulations of 2 m temperature (2mT), 2 m relative humidity (2mRH), 10 m wind speed (10mWS), and near-surface PM_2.5_ concentration were compared for SY and the CLCC (Table 2). The U-U case is more accurate for the temperature simulation results in SY and the CLCC, with R and IOA above 0.93 and 0.91, respectively, but the simulation results are slightly higher. Its simulation of relative humidity is also more accurate, with R and IOA above 0.57 and 0.74 for SY and the CLCC, respectively, and better for SY, where the simulated value is slightly lower than the observed value. However, its simulation results for wind speed are relatively more overestimated, and the simulation for the CLCC is better than that for SY, with R and IOA reaching 0.50 and 0.27, respectively. The simulation results of the U-U case for near-surface PM_2.5_ concentrations showed that SY was overestimated, while the CLCC was slightly underestimated, and also the simulation for the CLCC was better than that of SY, with R and IOA reaching 0.58 and 0.76, respectively. The U-U case overestimates temperature and wind speed and underestimates relative humidity, which is consistent with the results of Liao et al. [10] and Lai et al. [12]. The WRF model still has some difficulties with wind simulation. Overall, the simulation results can reasonably reflect the actual atmospheric conditions and near-surface PM_2.5_ concentrations, and these good model properties provide a good basis for using the simulation results to investigate the physical processes behind the pollution events in the CLCC. It is worth noting that there are still biases in the model results compared to observations, which can be attributed to the initial weather and boundary conditions [61], as well as limitations of the physical and chemical schemes [62]. Since the focus of this study is on the variation of contaminant concentrations, the differences between simulations are of greater interest relative to the absolute values, and the WRF–Chem model used in this study is an appropriate tool [10].

## 3. Results and Analysis

### 3.1. Pollution Process of the CLCC from 1 to 5 January 2020

**Figure 3:**
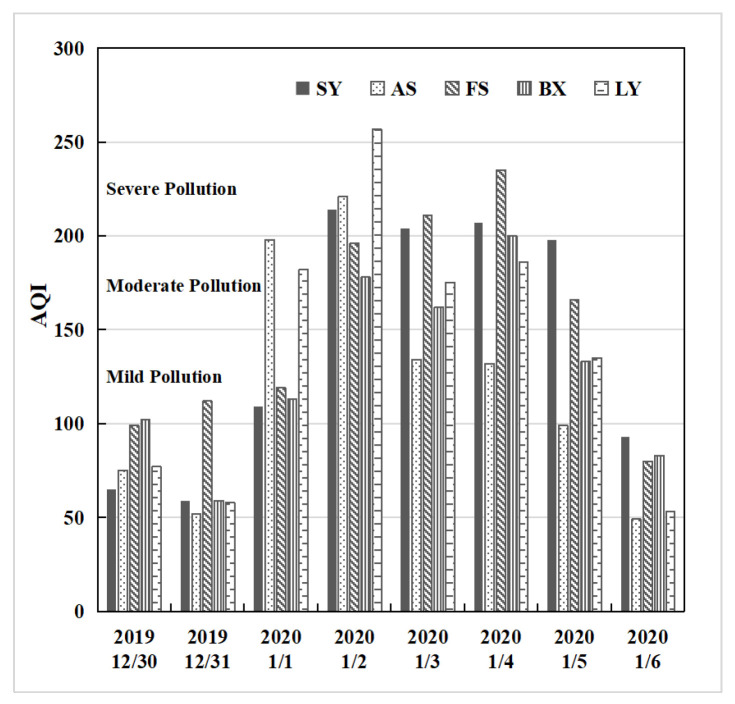
Daily average AQI observation results of cities in the CLCC from 30 December 2019 to 8 January 2020.

**Figure 4:**
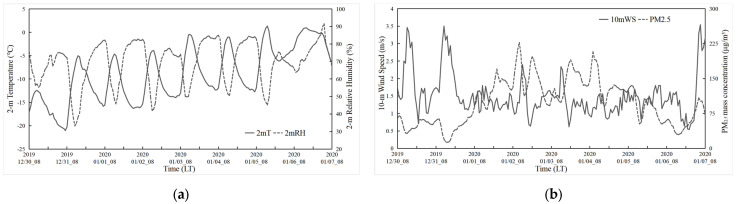
Time series of observed hourly mean (**a**) 2 m temperature (°C), 2 m relative humidity (%), and (**b**) 10 m wind speed (m/s) and near-surface PM_2.5_ mass concentration (μg/m^3^) at the CLCC from 30 December 2019 to 7 January 2020.

A widespread pollution event took place in the CLCC from 1 to 5 January 2020, accompanied by poor meteorological diffusion conditions characterized by weak winds, strong thermal stability, and a shallow planetary boundary layer [40]. The daily average AQI of the CLCC is given in Figure 3. From 30 to 31 December 2019, the AQI in the CLCC was mostly good, with some cities experiencing mild pollution. From 1 to 5 January 2020, except for AS AQI of 99 on the 5th, there was a widespread and sustained process of mild to moderate pollution in the CLCC and severe pollution in LY. For this pollution process, Figure 4 shows the time series of average meteorological elements and particulate matter concentrations in the CLCC. The temperature and relative humidity were lower, and the wind speed was higher in the CLCC on 30–31 December 2019, during which the PM_2.5_ concentrations were lower. This was mainly due to the strong northwest/west winds controlled by the northeastern low-pressure system and the entry of cold air masses [40]. This was followed by a sustained widespread pollution process with hourly PM_2.5_ concentrations exceeding 75 μg/m^3^ from 1 to 5 January 2020, as temperature and relative humidity increased and wind speed decreased. The air quality in the CLCC gradually improved from 6 January, partly due to the onset of precipitation [40].

### 3.2. The Impacts of Urbanization on Regional Atmospheric Diffusion Capacity

The five cities in the CLCC are at the center of a highly urbanized region in the Liaoning Province, and urbanization will cause significant changes in the surface albedo, heat capacity, and heat transfer rate of the urban subsurface, with consequent changes in their absorption and reflection of shortwave and longwave radiation, leading to changes in the surface sensible and latent heat fluxes, and these changes will directly affect the surface air temperature in urban areas [12], which in turn will affect the structure of the atmospheric boundary layer [33]. In addition, urbanization changes land use from a natural surface to an impermeable concrete surface, which increases the surface roughness that drags the atmosphere. Moreover, the high temperature and turbulence of the urban subsurface affect the winds in the atmospheric boundary layer by changing the horizontal pressure gradient and vertical diffusivity [63], leading to increased low-level atmospheric convergence in urban areas. This increases atmospheric instability, which can transport water vapor upwards, favoring the onset of convection and the transport of water vapor to the upper atmosphere, leading to a reduction in surface water vapor [64].

**Figure 5:**
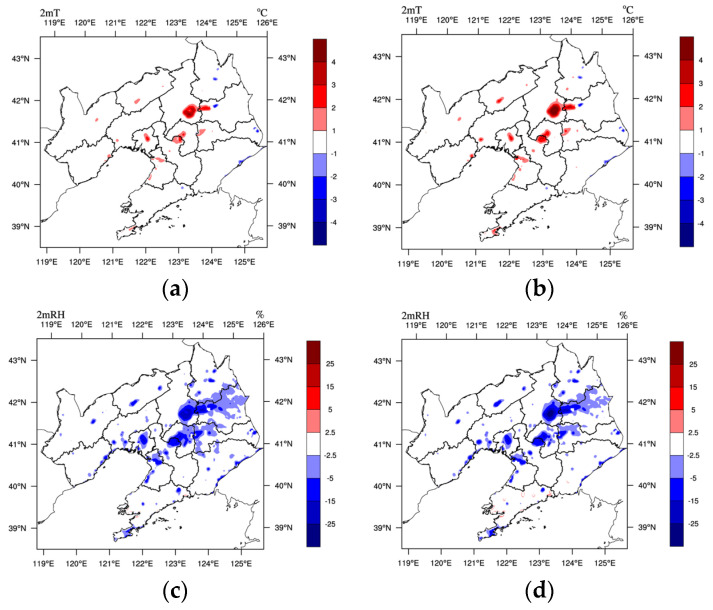

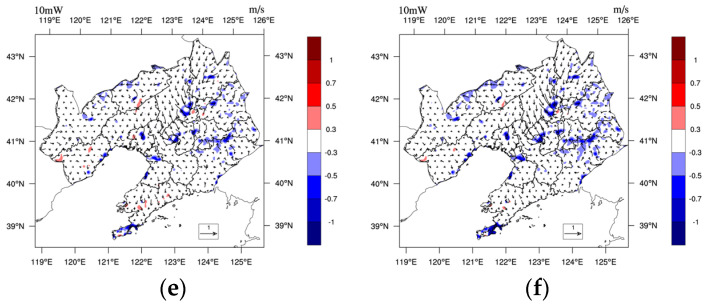
Horizontal distribution of the difference of mean (**a**,**b**) 2 m temperature (°C), (**c**,**d**) 2 m relative humidity (%), (**e**,**f**) 10 m wind speed (m/s, colored plot) and wind field (vector plot) during the pollution period 1–5 January 2020 between (**a**,**c**,**e**) M-B and U-B, (**b**,**d**,**f**) M-B and U-U, respectively.

The distribution of changes in 2mT, 2mRH, and 10 m wind due to the LU and LUUC effects are given in Figure 5. Both the LU and LUUC effects result in a significant increase in 2mT and a significant decrease in 2mRH and 10mWS in Liaoning, with a distribution of changes similar to that of urban LU (Figure 2). The LU and LUUC effects resulted in a 21.7% and 9.9% decrease in surface albedo and a 62.9% and 89.7% increase in sensible heat flux (SH), respectively, which in turn led to a 1.98 °C (40.1%) and 2.64 °C (53.9%) increase in 2mT and a 13.08% (25.1%) and 14.88% (28.5%) decrease in 2mRH in the CLCC. The LU and LUUC effects resulted in northerly wind differences in Liaoning, with 10mWS decreasing by an average of 0.30 m/s (20.5%) and 0.41 m/s (28.1%), respectively. The increase in surface temperature and decrease in relative humidity and wind speed due to urbanization are consistent with the findings of Lai et al. [12] and Dai et al. [13] in the Pearl River Delta region.

Daytime (08-16, LST) and nighttime (17-07) are defined according to the variation of surface shortwave radiation from the CLCC. Compared to the daytime warming (1.98 °C and 2.64 °C), the LU and LUUC effects resulted in greater warming at 2mT at night, 2.31 °C and 3.04 °C, respectively. The reduction in 10mWS due to the LU and LUUC effects occurs mainly at night (−0.44 and −0.60 m/s), with little reduction during the daytime (−0.06 and −0.08 m/s). On the other hand, the reduction of 2mRH due to the LU and LUUC effects did not differ significantly during the daytime (−13.31% and −14.29%) and at night (−12.95% and −15.24%). As a whole, the LUUC effect causes significantly greater changes than the LU effect, with the UC effect playing the same role as the LU effect, but weaker. 

Changes in surface temperature are influenced by the energy and radiation balance. To further explain changes in surface meteorological elements, surface energy is introduced for analysis [2,65]. The surface energy balance of the urban subsurface can be expressed as Rn = SH + LH + G0, where Rn is the net radiative flux, the main source of energy used to heat the atmosphere and the surface in the surface radiation balance, including net shortwave radiation (SW) and net longwave radiation (LW) at the surface. The sensible heat flux (SH) reflects the heat exchange between the surface and the atmosphere in the form of turbulence. The latent heat flux (LH) is mainly influenced by surface water vapor evaporation and vegetation transpiration, and reflects the hydrothermal exchange between the Earth and the atmosphere. The ground heat flux (G0) accounts for the energy gained or lost as the surface warms or cools [65]. Normally, G0 is negative during the daytime; the ground is dominated by heat storage, a large amount of solar radiation is absorbed and captured by buildings, SH is positive, and heat is transferred from the ground to the atmosphere. G0 is positive at night when the surface is mainly exothermic, SH is negative, and heat is transferred from the atmosphere to the surface. 

**Figure 6:**
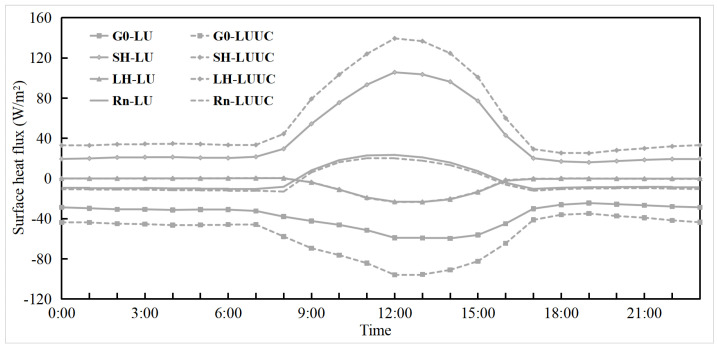
Daily variation of mean surface heat flux in the CLCC during the pollution period 1–5 January 2020 caused by the LU and LUUC effects.

The changes In surface heat fluxes In the CLCC due to the LU and LUUC effects are shown in Figure 6 and Table 3. Both the LU and LUUC effects cause the Rn at the surface to increase during the daytime and decrease at night, with a slight overall decrease (−1.57 and −3.56 W/m^2^, respectively). With the LU and LUUC effects, the increase in SW during the daytime is slightly smaller than the growth of LW spending (negative) throughout the day. The UC effect enhances the increase in net SW at the surface, while the increase in LW spending is greater, so the LUUC effect adds slightly less to Rn than the LU effect. Changes in Rn significantly increase SH release and surface heat storage (negative G0) in urban areas [12], and the increase is greater during the daytime, with the UC and LU effects playing the same role, and the UC effect enhancing the increase in SH and surface heat storage. The increased surface heat storage during the daytime releases more heat flux at night, which explains why there is more warming at night. In addition, the slow reduction of SH release in urban areas after sunset, and the allocation of surface heat storage released from cities to a relatively short atmospheric column at night (lower boundary layer height than during the daytime) [12] are partly responsible. The decrease in LH due to the LU and LUUC effects is similar and mainly during the daytime, which is mainly because of the reduction of local water vapor sources due to the surface impermeability caused by the LU effect. This also partly explains why the warming is weaker during the daytime than at night. The UC effect has little impact on LH changes. In summary, the LU and LUUC effects change the surface energy distribution relationship and increase the net SW at the surface during the daytime, which significantly enhances the SH transport and weakens the LH, and increases surface heat storage.

**Figure 7:**
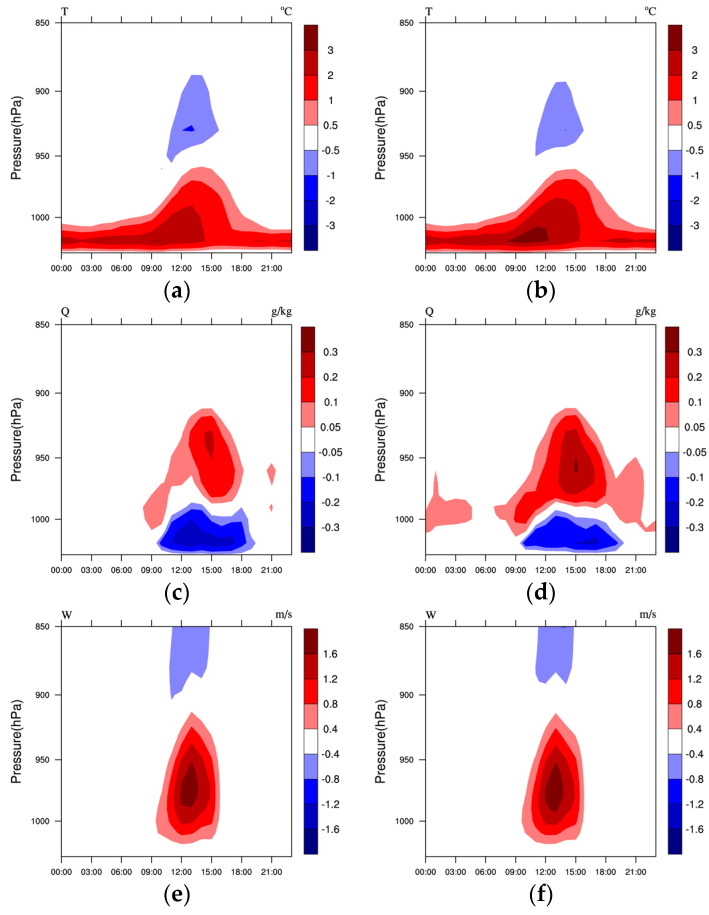
Time–height cross-section of the difference of mean (**a**,**b**) temperature (T, °C), (**c**,**d**) water vapor (Q, g/kg), and (**e**,**f**) vertical velocity (W, m/s) during the pollution period 1–5 January 2020 between (**a**,**c**,**e**) M-B and U-B, (**b**,**d**,**f**) M-B and U-U, respectively.

Figure 7 shows the LU and LUUC effects on mean temperature, water vapor, and vertical velocity with time and altitude during the pollution period in Shenyang (41.7352° N, 123.5100° E). Combined with the daily changes in surface heat fluxes in Figure 6, it can be seen that both the LU and LUUC effects lead to warming of the near-surface layer throughout the day in Shenyang, with more warming at night. From 06:00, the warming spread to the upper layers with the sunrise and reached its maximum at about 13:00. The affected height could reach about 950 hPa (about 500 m), while the temperature decreased above 950 hPa. The inversion in the U-U case occurred mainly around 03:00 and 06:00. The inversion is weakened by the near-surface warming that begins at 06:00 due to the LU and LUUC effects, leading to increased atmospheric instability and favoring the diffusion of pollutants. Accompanying the temperature rise in the near-surface layer and its upward expansion, a significant convective increase below about 900 hPa was observed starting at 09:00 (Figure 7e,f), reaching a maximum at 13:00, when the warming was most pronounced, and disappearing after 17:00 at sunset. This is due to the enhanced absorption of SW by the urban subsurface during the daytime caused by the LU and LUUC effects, and the bottom-up radiation is reflected multiple times by the surfaces of buildings in the city, creating the effect of urban radiation traps. At the same time, most of the energy absorbed in urban areas is used as SH to heat the atmosphere, lifting the airflow near the top of the boundary layer and producing updrafts. There is a sinking enhancement above 900 hPa, which is the reason for the temperature decrease above 950 hPa. The LU and LUUC effects lead to a decrease in near-surface water vapor during the daytime, mainly between 09:00 and 19:00, due to differences in the vertical upward airflow, resulting in the transport of surface water vapor to higher altitudes and an increase in the upper layers. This is consistent with the findings of Chen et al. [1] and Lai et al. [12]. The UC effect also leads to an increase in water vapor at night, which is mainly due to the fact that the city still maintains higher temperatures at night, and the water vapor in the air cannot condense, thus increasing water vapor at night.

**Figure 8:**
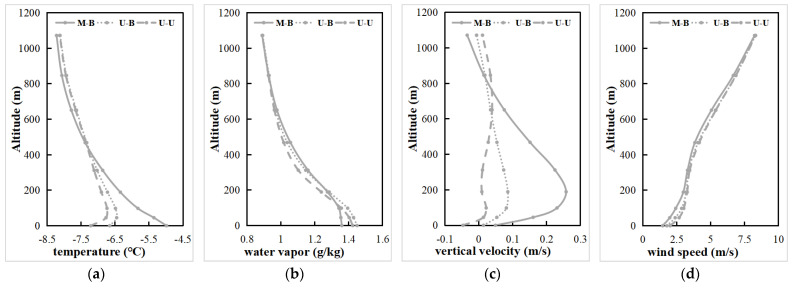
Profiles of the mean (**a**) potential temperature (°C), (**b**) water vapor (g/kg), (**c**) vertical velocity (m/s), and (**d**) horizontal wind speed (m/s) during the pollution period 1–5 January 2020 in the CLCC simulated by three cases.

As can be seen from the simulated CLCC temperature profiles for the three sets of experiments (Figure 8a), in the U-B and U-U experiments where the LU was not updated, there was an inversion in the lower atmosphere that was not conducive to vertical diffusion of the pollutants. In contrast, the LU and LUUC effects cause significant increases in surface and near-surface temperatures in the CLCC, increasing atmospheric instability. The decrease in temperature above about 450 m may be due to convective upwelling caused by near-surface heating. The LU and LUUC effect decreases the water vapor content below about 100 m in the near-surface layer of the CLCC and increases it above 200 m (Figure 8b), again probably related to uplift. The UC effect, on the other hand, leads to a slight increase in both temperature and water vapor, mainly as a result of temperature and water vapor trapping by the complex UC [11]. Because of the LU and UC effects, which increase the near-surface temperature, and the blocking effect of urban buildings, the LUUC effect shows a stronger vertical upward motion from the ground to about 600 m in the CLCC (Figure 8c) (thermal effect). The UC effect also leads to an increase in vertical upward motion, but not as significant as the LU effect. Additionally, the LU and UC effects lead to the reduction of the horizontal wind speed due to the drag-damping effect of the wind by the dynamic effect of the urban buildings (Figure 8d). Moreover, the increase in vertical velocity in the city converts the horizontal kinetic energy of the airflow into vertical kinetic energy, which reduces the horizontal wind speed. Overall, although the LU effect leads to a decrease in near-surface horizontal wind speed, the increase in temperature and vertical motion leads to an increase in atmospheric instability, which may increase the ADC of the urban. The UC effect causes a slight increase in vertical velocity and a decrease in horizontal wind speed, which may not have a significant effect on the ADC.

**Figure 9:**
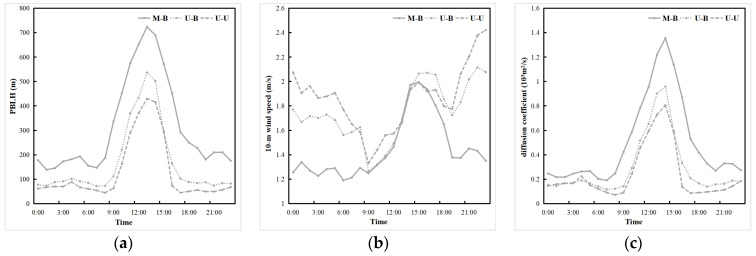
Daily variation of the mean (**a**) boundary layer height (m), (**b**) 10 m wind speed (m/s), and (**c**) diffusion coefficient (10^3^·m^2^/s) during the pollution period 1–5 January 2020 in the CLCC simulated by three cases.

Stronger surface thermal effect favors increased atmospheric instability, which promotes the development of convection, resulting in more rapid boundary layer development and, thus, a higher boundary layer [33]. The top of the atmospheric boundary layer separates the boundary layer from the free troposphere, so the boundary layer height (BLH) describes the vertical extent of mixing processes generated within the atmospheric boundary layer and is a key variable affecting pollutant transport and diffusion [12,63]. The daily variation of the BLH in the CLCC shows that the BLH is lower at night and reaches its maximum at 13:00 (Figure 9a). Both the LU and LUUC effects resulted in significant increases in the BLH of 214.91 m (41.7%) and 276.73 m (53.7%) during the daytime, and 105.05 m (55.1%) and 129.70 m (68.0%) at night, respectively. This is attributed to the Increase In the Intensity of boundary layer turbulence and convective instability caused by the temperature increase due to the LU and LUUC effects, which favors the development of the boundary layer. The UC effect plays a similar role as the LU effect. The higher BLH growth rate at night than during daytime may be mainly due to the greater warming at night. The variation of 10mWS in the CLCC due to LU and LUUC effects was dominated by decreases (Figure 9b), with wind speed decreases of 0.30 m/s (20.5%) and 0.41 m/s (28.1%), respectively, but the decreases occurred mainly at night (−0.44 m/s and −0.60 m/s). The decrease in wind speed at night is not conducive to the diffusion of pollutants in urban areas and may contribute to air pollution events. This finding is consistent with previous studies [1,32]. 

The LU and LUUC effects in this study result in a greater change in vertical velocity than horizontal velocity, which means that the thermal effect is significantly greater than the dynamic effect, increasing the ADC. To test this idea, diffusion coefficients are introduced to further discuss the variation of vertical turbulent mixing processes that play an important role in boundary layer dynamics [63]. The diffusion coefficient takes into account the combined effects of vertical and horizontal mixing and can be used to describe the intensity of turbulent mixing and indicate the ability of the atmosphere to disperse air pollutants. In this study, the product of the BLH (m) and 10mWS (m/s) was defined as the diffusion coefficient (DC = BLH × 10mWS, 10^3^·m^2^/s) to characterize the excellent diffusion conditions. Both the LU and LUUC effects increased the diffusion coefficient by 0.21 × 10^3^·m^2^/s (41.4%) and 0.26 × 10^3^·m^2^/s (51.6%), respectively. Although the increase in the diffusion coefficient is numerically larger during the daytime than at night, the rate of increase is slightly higher at night, which is mainly due to the larger increase rate in the BLH at night caused by the LU and LUUC effects, which increases the diffusion coefficient despite the decrease in wind speed. Therefore, this finding also proves that the LU and LUUC effects lead to a greater thermal than dynamic effect, and although the nighttime wind speed decreases, it still leads to an overall increase in the ADC. 

### 3.3. The Impacts of Urbanization on Regional PM_2.5_

Previous research has shown that air pollution is closely related to meteorological factors and that pollutant concentrations are strongly influenced by meteorological conditions when determining pollutant emission inventories [5]. The above analysis shows that the LU and LUUC effects can lead to significant changes in meteorological conditions and the ADC within the atmospheric boundary layer, which in turn can affect the distribution of air pollutants. The pollution case selected for this study is in winter, and the main air pollutant in the CLCC is PM_2.5_. The effects of LU and LUUC on regional air quality are studied using PM_2.5_ as an example.

**Figure 10:**
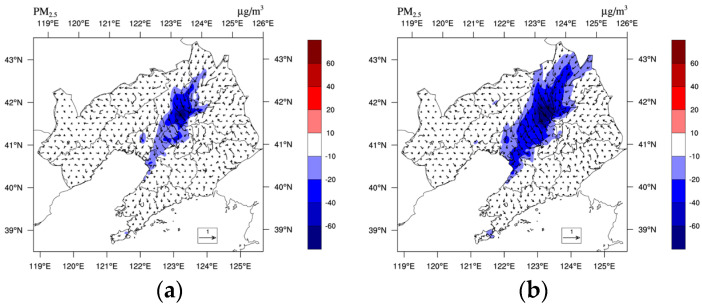
Horizontal distribution of the difference of mean near-surface PM_2.5_ concentration (μg/m^3^) during the pollution period 1–5 January 2020 between (**a**) M-B and U-B, (**b**) M-B and U-U, respectively.

**Figure 11:**
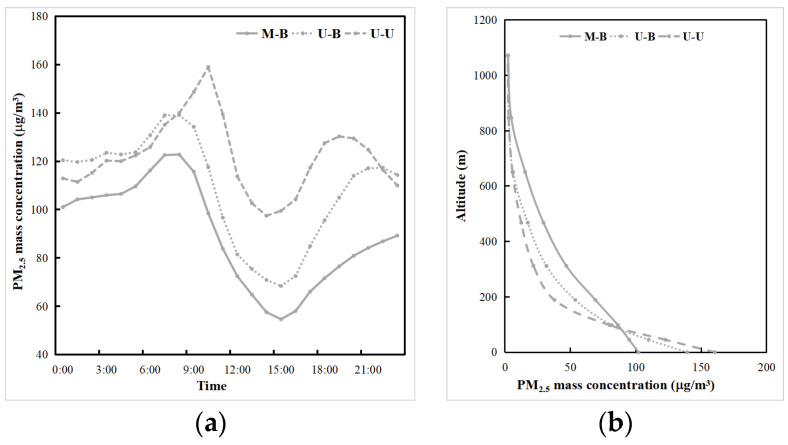
Daily variation of the mean (**a**) near-surface PM_2.5_ concentration (μg/m^3^) and (**b**) profiles of mean PM_2.5_ concentration (μg/m^3^) during the pollution period 1–5 January 2020 in the CLCC simulated by three cases.

As shown in Figure 10, the LU and LUUC effects resulted in significant reductions in near-surface PM_2.5_ concentrations in the CLCC, centered on Shenyang, with average PM_2.5_ concentrations decreasing by 18.77 μg/m^3^ (20.9%) and 32.03 μg/m^3^ (35.7%), respectively, during the pollution periods. The increased ADC due to LU and LUUC effects facilitates vertical diffusion and dilution of PM_2.5_ within the boundary layer [12]. The daily variation process of near-surface PM_2.5_ concentration at CLCC in winter starts to decrease after reaching a maximum in the morning, and then increases again after reaching a minimum in the afternoon (Figure 11a). This is due to the increase in PM_2.5_ concentrations caused by the combined effect of morning inversions and low BLH. In addition, the increase in particulate matter caused by traffic emissions during the morning rush hour is also a cause. As the BLH and the diffusion coefficient increase during the daytime, the pollutant concentration gradually decreases, and the minimum value appears corresponding to the time when the BLH reaches its maximum (Figure 9a). The concentration increases again in the evening with the decrease of the BLH and the evening peak emission. The diurnal variation of the U-B experiment without renewed land use was similar to that of the M-B experiment, with higher concentrations, especially at night. The LU effect resulted in an overall reduction in surface PM2.5 concentrations, but the reduction was less during daytime (14.23 μg/m^3^, 17.6%) than at night (21.49 μg/m^3^, 22.6%). In contrast, the morning peak concentration of the U-U experiment was delayed, reached a maximum before noon, and showed another small peak at 19:00. Compared to the M-B experiment, the warming caused by the LUUC effect resulted in an earlier morning peak concentration and a weaker diurnal trend, leading to a greater reduction in diurnal near-surface PM_2.5_ concentrations (41.81 μg/m^3^, 51.7%). Additionally, the more complex UC scheme (BEP) causes stronger diffusion and dilution due to the influence of urban turbulence at different levels.

Figure 11b shows the profiles of mean PM_2.5_ concentration during the pollution periods in the CLCC, where the PM_2.5_ concentration decreases near the surface but increases above due to the LU and LUUC effects. This is mainly due to the increase in the ADC, which results in more PM_2.5_ being transported into the air above 100 m. The ratio of PM_2.5_ concentrations above 500 m to below 500 m increased by 3.0% and 3.7% in the CLCC due to the LU and LUUC effects, respectively. The change in this ratio suggests that the LU and LUUC effects can redistribute pollutants from the lower urban troposphere to higher altitudes, which is consistent with the findings of Zhong et al. [66].

**Figure 12:**
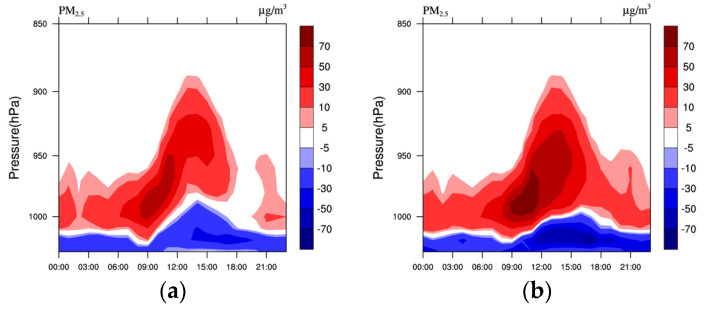
Time–height cross-section of the difference of average PM_2.5_ concentration (μg/m^3^) during the pollution period 1–5 January 2020 in Shenyang between (**a**) M-B and U-B, (**b**) M-B and U-U, respectively.

From the time–height cross-section of PM_2.5_ concentrations in Shenyang (Figure 12), it can be seen that the LU and LUUC effects, although having little effect on the nighttime vertical velocity (Figure 7e,f) and the decrease in horizontal wind speed (Figure 8d), increase the temperature (Figure 7a,b) and the BLH (Figure 10a), which leads to an increase in the ADC at night (Figure 9c), resulting in a decrease in PM_2.5_ concentrations in the near-surface layer at night and an increase in the upper layers. After sunrise, as surface temperatures begin to rise due to the LU and LUUC effects (Figure 7a,b), the atmosphere above becomes unstable and turbulent motions increase the BLH (Figure 9a). The increased BLH causes PM_2.5_ and water vapor to be elevated, reaching a maximum in the late afternoon when the atmospheric diffusion capacity increases significantly (Figure 9c), and the transport of PM_2.5_ and water vapor from lower levels to higher altitudes leads to a decrease In PM_2.5_ and water vapor concentrations in the near-surface layer and an increase in the upper layer (Figure 12). Overall, the LU and LUUC effects on surface PM_2.5_ concentrations were negative due to altered thermal processes, but increased at higher altitudes, consistent with the findings of others [1,12,14].

## 4. Conclusions

In this study, the effects of land-use change (LU) and urban canopy (UC) on the atmospheric environment of the Central Liaoning city cluster (CLCC) were investigated by three sets of simulation experiments using the WRF–Chem model. The simulation experiments used different land-use data and UC schemes. We assume the same anthropogenic emissions in the simulations to focus on the effects of LU and both LU and UC (LUCC) on boundary layer meteorological conditions and air pollutant concentrations. The LU and LUUC effects were isolated by comparing the results of M-B and U-B, and M-B and U-U experiments, respectively. A large-scale pollution process in the CLCC from 1 to 5 January 2020 was selected, and model validation with observed data showed that the model could reproduce the meteorological and chemical conditions well. 

Both the LU and LUUC effects change the surface energy distribution by causing a decrease in surface albedo, an increase in sensible heat flux, and surface roughness in the CLCC, which lead to an increase in 2 m temperature, 2 m relative humidity, and 10 m wind speed in the CLCC, with a distribution of changes similar to that of urban LU. The LU and LUUC effects resulted in 2 m temperature increases of 2.31 °C and 3.04 °C at night, respectively, compared to higher daytime warming (1.98 °C and 2.64 °C). This is mainly due to the fact that increased surface heat storage during the daytime releases more heat at night, the decrease in latent heat flux occurs mainly during the daytime, the slow decrease in sensible heat flux released after sunset in urban areas, and the distribution of surface heat storage released from cities into a relatively short atmospheric column at night (with a lower boundary layer height (BLH) than during daytime). Taking Shenyang as an example to make a time–height cross-section, it is also found that both the LU and LUUC effects lead to a predominantly warming near-surface temperature in Shenyang throughout the day, with a weakening of the inverse temperature at night and early morning. During the daytime, the thermal effect lifts the atmosphere and increases instability, which is conducive to the diffusion of pollutants. The uplift caused by the LU and LUUC effects leads to a decrease in near-surface water vapor during the daytime and an increase in the upper layers. In addition, the higher urban temperatures at night prevent water vapor from condensing, leading to an increase in water vapor at night. 

Thermal effects from the LU and LUUC effects increase surface and near-surface air temperatures, leading to enhanced vertical upward motion and upward transport of water vapor in the near-surface layer, as well as reduced temperatures and increased water vapor in the upper layers. Dynamic effects from the LU and LUUC effects reduce horizontal wind speeds. Although the LU and LUUC effects lead to a decrease in near-surface horizontal wind speed, the increased temperature and vertical motion lead to an increase in atmospheric instability and a significant increase in the BLH, resulting in an increase in the all-day diffusion coefficient and the atmospheric diffusion capacity (ADC). The LU and LUUC effects in this study resulted in a greater thermal effect than a dynamic effect. 

The LU and LUUC effects lead to a decrease in near-surface PM_2.5_ concentrations in the CLCC by 18.77 μg/m^3^ (20.9%) and 32.03 μg/m^3^ (35.7%), respectively, which are influenced by the meteorological conditions within the altered boundary layer and the ADC. The reduction in near-surface PM_2.5_ concentrations due to the LU effect is stronger at night than during the daytime. The LUUC effect, on the other hand, leads to a stronger reduction in near-surface PM_2.5_ concentrations during the daytime, and the more complex BEP scheme leads to stronger diffusion and dilution due to the influence of urban turbulence within different layers. Due to the effects of LU and LUUC, the ratio of PM_2.5_ concentration above 500 m to below 500 m in the CLCC increased by 3.0% and 3.7%, respectively. This suggests that the LU and LUUC effects have a negative impact on near-surface PM_2.5_ concentrations due to altered thermal and dynamical processes, but redistribute pollutants from the lower urban troposphere to higher altitudes.

The above results suggest that the atmospheric environment is sensitive to LU, but the intrinsic mechanisms need further investigation. Due to the rapid urban development, the urban coverage in the USGS and MODIS land-use data sets supplied with the model may be small compared with the actual situation, so the MODIS 2018 land-use data set is introduced in this work to obtain a more realistic simulation. We also performed a simulation analysis for an entire month with similar results, but with less variation in PM_2.5_ concentrations, so this study was performed for a single pollution event with the aim of highlighting the effects of LU and LUUC. There are some limitations in the numerical experiment of this study; for example, the parameters used in the BEP are taken from the model, such as building height, road width, and surface albedo. In future studies, the parameters can be further modified according to the actual situation in the CLCC area. In addition, the urban fraction in the default BEP scheme is a fixed value of 0.95 assigned to all central urban and peri-urban networks, whereas in actuality, the urban fraction is not evenly distributed, so the fixed urban fraction value does not represent regional distribution differences well and needs further refinement. Furthermore, in addition to LU, anthropogenic emissions due to urbanization, anthropogenic heat change, and aerosol radiation interactions are also important for the atmospheric environment. Research suggests that aerosols scatter solar shortwave radiation and reduce atmospheric temperatures. The elevated upper-level aerosols will cool the upper atmosphere, while the LU effect causes warming in the near-surface layer; there will be a positive feedback mechanism that leads to a more unstable atmosphere over the city, which needs to be investigated in the future.

## Figures and Tables

**Table 1:** Simulation experiments.

Experiment	Land-Use Data Set	Urban Canopy Scheme
U-U	USGS	UCM
M-B	MODIS 2018	BEP
U-B	USGS	BEP

**Table 2:** Statistical evaluation of simulation results at SY and the CLCC from 00:00 31 December 2019 to 08:00 7 January 2020.

Region	Variables	MB	GE	RMSE	R	IOA
SY	2 m temperature (°C)	1.85	2.54	3.09	0.93 **	0.91
	2 m relative humidity (%)	1.95	8.09	10.27	0.71 **	0.81
	10 m wind speed (m/s)	1.44	1.53	1.94	0.40 **	0.19
	Near-surface PM_2.5_ (µg/m^3^)	27.52	55.65	65.97	0.48 **	0.63
CLCC	2 m temperature (°C)	1.24	2.10	2.42	0.95 **	0.94
	2 m relative humidity (%)	−0.99	8.62	10.73	0.57 **	0.74
	10 m wind speed (m/s)	0.90	0.99	1.28	0.50 **	0.27
	Near-surface PM_2.5_ (µg/m^3^)	−1.62	31.20	41.81	0.58 **	0.76

Note: ** indicates that the values have passed the significance test at the 0.01 significance level.

**Table 3:** Variation of surface heat flux (W/m^2^) in the CLCC due to the LU and LUUC effects.

Effect	SW			LW			Rn		
	Mean	Daytime	Nighttime	Mean	Daytime	Nighttime	Mean	Daytime	Nighttime
LU	9.36	24.96	0.00	−10.93	−13.36	−9.47	−1.57	11.60	−9.47
LUUC	9.77	26.07	0.00	−13.33	−17.26	−10.97	−3.56	8.81	−10.97
**Effect**	**SH**			**G0**			**LH**		
	Mean	Daytime	Nighttime	Mean	Daytime	Nighttime	Mean	Daytime	Nighttime
LU	40.49	75.39	19.55	−37.02	−49.43	−29.57	−4.89	−13.00	−0.02
LUUC	57.73	101.40	31.53	−57.30	−75.88	−46.15	−4.87	−12.88	−0.06

## Data Availability

Data used in this paper may be provided by Dongdong Wang (wangdongdong@iaesy.cn) upon request.
